# Update on leukotriene receptor antagonists in preschool children wheezing disorders

**DOI:** 10.1186/1824-7288-38-29

**Published:** 2012-06-26

**Authors:** Silvia Montella, Marco Maglione, Sara De Stefano, Angelo Manna, Angela Di Giorgio, Francesca Santamaria

**Affiliations:** 1 Department of Pediatrics, Federico II University, Via Sergio Pansini, 5, Naples, 80131, Italy

**Keywords:** Leukotriene receptor antagonists, Asthma, Preschool children, Wheezing, Bronchiolitis

## Abstract

Asthma is the most common chronic disease in young children. About 40% of all preschool children regularly wheeze during common cold infections. The heterogeneity of wheezing phenotypes early in life and various anatomical and emotional factors unique to young children present significant challenges in the clinical management of this problem. Anti-inflammatory therapy, mainly consisting of inhaled corticosteroids (ICS), is the cornerstone of asthma management. Since Leukotrienes (LTs) are chemical mediators of airway inflammation in asthma, the leukotriene receptor antagonists (LTRAs) are traditionally used as potent anti-inflammatory drugs in the long-term treatment of asthma in adults, adolescents, and school-age children. In particular, montelukast decreases airway inflammation, and has also a bronchoprotective effect. The main guidelines on asthma management have confirmed the clinical utility of LTRAs in children older than five years. In the present review we describe the most recent advances on the use of LTRAs in the treatment of preschool wheezing disorders. LTRAs are effective in young children with virus-induced wheeze and with multiple-trigger disease. Conflicting data do not allow to reach definitive conclusions on LTRAs efficacy in bronchiolitis or post-bronchiolitis wheeze, and in acute asthma. The excellent safety profile of montelukast and the possibility of oral administration, that entails better compliance from young children, represent the main strengths of its use in preschool children. Montelukast is a valid alternative to ICS especially in poorly compliant preschool children, or in subjects who show adverse effects related to long-term steroid therapy.

## Introduction

Asthma is the most common chronic disease of the airways in young children [1]. About 40% of all preschool children regularly wheeze during common cold infections. Although about two-thirds of these children lose their symptoms after the age of six years, the disease places a considerable burden on the child, the child’s family, and society because of the high prevalence and lack of good treatment control [2]. Moreover, the heterogeneity of wheezing phenotypes early in life presents significant challenges in the clinical management of this problem.

Anti-inflammatory therapy is the cornerstone of asthma management. Studies of the efficacy of inhaled corticosteroids (ICS) in preschool children have given conflicting results. Some studies show that ICS are effective in improving symptoms and lung function [3-8], although safety data are worrisome [9], while others find no effect at all on the prevention of progression to established asthma [10-12]. The most plausible explanation for these differences is that there is no single wheezing phenotype in young children, since the disease may be provoked by respiratory viruses, allergens, exercise and exposure to smoke or other pollutants [13]. In addition to this, there are various anatomical, physiological and emotional factors unique to young children, especially infants, that result in significant difficulties and challenges to inhalation therapy [14]. All these troubles can be easily overcome by oral drug administration.

The leukotriene receptor antagonists (LTRAs) are traditionally used as anti-inflammatory drugs in the long-term treatment of asthma in adults, adolescents, and school-age children [15-17]. This review summarizes the most recent development on the use of LTRAs in the treatment of preschool wheezing disorders.

## Leukotrienes mechanism of action and biological effects

**Figure 1:**
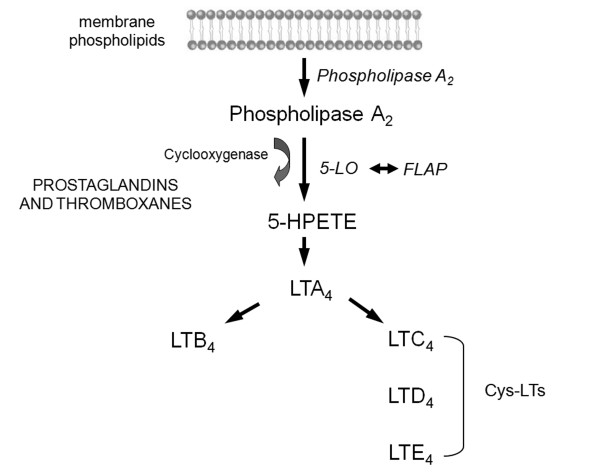
**Leukotriene biosynthesis.** LT, leukotriene; 5-LO, 5-lipoxygenase; FLAP, five lipoxygenase activating protein; 5-HPETE, 5- hydroperoxyeicosatetraenoic acid; Cys-LTs, cysteinyl leukotrienes.

Leukotrienes (LTs) are chemical mediators of airway inflammation in asthma, produced from membrane phospholipids via the 5-lipoxygenase pathway of the arachidonic acid cascade (Figure 1) [18]. After activation by mechanical, chemical, or physical stimuli, cytosolic phospholipase A2 translocates to the membrane to liberate membrane-bound arachidonic acid. Free arachidonic acid either can be converted by the cyclooxygenase pathway to form prostanoids (prostaglandins, prostacyclin and thromboxane) or, alternatively, can follow the 5-lipoxygenase pathway to form LTs. In combination with the 5-lipoxygenase activating protein, the 5-lipoxygenase enzyme acts on arachidonic acid to form the 5-hydroperoxyeicosatetraenoic acid, which is then converted to the nonpeptide leukotriene LTA_4_. LTA_4_ can then be converted to the cysteinyl leukotrienes (CysLTs) LTC_4_, LTD_4_ and LTE_4_ which provoke bronchial and vascular constriction and therefore play a central role in the pathophysiology of asthma [18].

The CysLTs are synthesized by several cells, including neutrophils, eosinophils, mast cells, alveolar macrophages, epithelial cells and vascular endothelial cells, which are present or recruited to the lung when airways inflammation occurs. The production of LTs by these cells depends on the selective expression of the enzymes involved in the metabolic pathway. LTA_4_ may be directly released into the extracellular environment and there be metabolized by other cells (transcellular biosynthesis). This pathway results in LTB_4_ production by bronchial epithelial cells, despite 5-lipoxygenase enzyme is absent [18].

Due to its activity in cellular recruitment and activation, LTB_4_ is an important proinflammatory mediator, also involved in the stimulation of the interleukin biosynthesis by T lymphocytes and monocytes, with consequent increase in vascular permeability and mucous production [19]. Conversely, CysLTs act on the smooth muscle cells, inducing a constrictive effect much stronger than that determined by methacoline or histamine. Furthermore, they are potent in eliciting bronchoconstriction, mucous production and vasodilatation, and may enhance the airways hyperresponsiveness that is characteristic of the asthmatic disease [20].

CysLTs receptor antagonists bind the LTD_4_ receptor and prevent the interaction between the receptor and its physiological ligands [21]. Due to this receptor obstruction, LTs cannot activate the signal transduction that leads to bronchoconstriction. Among LTRAs, zafirlukast, pranlukast and montelukast have been approved in several countries for asthma treatment of adults and children. Montelukast (MK-0476), is the most specific and powerful LTs receptor antagonist, and is the only LTRA that has been approved for preschool children use in several countries including Italy. In addition to its use as controller drug in allergic and viral-induced asthma, it decreases also bronchial hyperreactivity and prevents bronchial obstruction induced by physical activity at preschool and school age [22,23].

CysLTs production is greatly increased in allergen- or exercise-induced asthma, particularly during exacerbations [24]. Children with steroid-naïve mild persistent, severe persistent and unstable asthma have high levels of exhaled CysLTs [25]. Moreover, CysLTs and LTB_4_ are significantly increased also in children with mild and moderate to persistent asthma treated with low or high doses of inhaled ICS [26].

CysLTs activate type 1 and type 2 leukotriene receptors (CysLT1 and CysLT2) on cell membranes [27]. CysLT1 receptors are localized primarily on pulmonary smooth muscle cells. Activation of these receptors by CysLT leads to decreased activity of respiratory cilia, increased mucous secretion, increased venopermeability and promotion of eosinophils into the airways [28]. This, in turn, induces airway smooth-muscle proliferation and may play a role in the development of airway remodeling. Moreover, CysLTs are the most potent bronchoconstrictive agents discovered, being 100–1000 times more potent than histamines [29].

## Scientific evidences about LTRAs in preschool children

### Monotherapy with montelukast

Only few short- or long-term randomized controlled trials (RCTs) with LTRAs have been conducted in preschool age.

#### Short-term monotherapy

A study of asthmatic children aged 2–5 years demonstrated that administration of 5 mg/die of montelukast for two days protects against cold air-induced bronchial hyperreactivity [30]. This finding was later confirmed in patients who showed a significant decrease in methacoline-induced bronchial reactivity after a four-weeks treatment with montelukast [22].

In atopic children with asthma, monotherapy with montelukast for 28 days was reported to be effective for reducing airway resistance and bronchial inflammation [31], and for improving lung function and symptoms score [32]. Finally, an Australian RCT conducted in children with mild intermittent asthma, demonstrated that a short course of montelukast, introduced at the first signs of an asthma episode, resulted in a significant reduction in acute health care resource utilization, symptoms, time off from school, and parental time off from work [33]. However, no significant effect on hospitalization rate, duration of symptoms, use of bronchodilators and systemic steroids was found, and finally, the number of symptom-free days, one of the primary outcomes of therapeutic efficacy, was not considered by the authors.

#### Long-term monotherapy

In 2001, a large multicenter, double-blind, international study on 689 preschool children with multi-trigger wheezing and persistent symptoms showed that daily montelukast given over three months is associated with more symptom-free days (34% *versus* 28% in the placebo group) [34]. In another study, Davies and coworkers found that patients aged 2 to 5 years with mild to moderate persistent asthma receiving long-term therapy with montelukast had similar rates of asthma-related health care resource utilization compared with those receiving usual care with cromolyn or ICS [35]. In 2005 the PREVIA study (PREvention of Viral Induced Asthma) showed that a 1-year course of montelukast in children with virus-induced asthma significantly reduces asthma exacerbations and the use of rescue medications [36]. Despite several criticisms, particularly regarding the number of symptom-free days that was not significantly different in the two groups (76% *versus* 73% in the placebo group, p = 0.059), and the use of systemic steroids that was not significantly reduced after treatment [37], the PREVIA study remains one of the few RCTs in preschool children with mild intermittent asthma that proves that prolonged treatment with montelukast reduces the consumption of ICS at a high rate (39.8%).

Studies that compared either short- or long-term treatment with LTRAs given as monotherapy to other antiasthma controller drugs in preschool children showed that montelukast is as effective as ICS. Szefler and coworkers evaluated the efficacy of montelukast *versus* budesonide in 349 children, mostly at preschool age, with mild persistent asthma, and found no differences between the two groups as far as drug tolerability, time to first additional anti-asthma medications and time to first severe exacerbation over a 52 weeks-study period [38]. Another small RCT assessed the efficacy of montelukast or fluticasone given as monotherapy *versus* placebo for three months [39]. Both drugs decreased asthma-like symptoms and improved the daily symptoms score, even though fluticasone appeared more effective than placebo. Nevertheless, the symptoms score also improved in the placebo group, suggesting that asthma-like symptoms, that are commonly associated with respiratory virus infections in preschool age, may spontaneously improve. Unfortunately, this study was conducted on a limited population and therefore the statistical power might be questioned [39].

In addition, the results of a RCT of preschool children, most of whom had positive asthma predictive indices, showed that the episodic use of either budesonide or montelukast for 7 days during an asthma exacerbation does not increase the proportion of symptom-free days or decrease oral corticosteroids and health care resources use [40]. Nevertheless, both drugs appeared effective in decreasing the severity of symptoms, particularly in cases with positive asthma predictive indices or with more severe clinical manifestations.

Finally, a retrospective analysis of 2034 asthmatic children treated with montelukast and fluticasone as monotherapy, showed that both drugs decrease rescue medications use and hospital visits [41]. In particular, in children aged 2 to 5 years, montelukast was associated to less emergency visits compared to subjects aged 6 to 14 years. This finding was taken by authors to conclude that the beneficial effects of LTRAs may be mainly observed in preschool children.

All the above findings indicate that LTRAs may be beneficial in preschool wheeze. However, since only two RCTs compared montelukast to ICS [39,40], more comparative studies of preschool children treated with LTRAs versus ICS should hopefully be promoted [42,43]. Likewise, the intermittent use of montelukast has been evaluated only in mild, or moderate-to severe intermittent wheeze [33,40], and this modality of treatment might be extended to children with severe recurrent or persistent symptoms.

### Combined therapy (montelukast *plus* inhaled steroids)

Combined therapy with montelukast and ICS in preschool children has been poorly investigated. One study of 194 children (22% aged 2 to 5 years) showed that montelukast added to the usual treatment with ICS reduced the risk of worsening asthma symptoms and unscheduled physician visits during the annual September asthma epidemic [44]. Another study had previously found that montelukast more effectively reduces acute asthma episodes if started before the viral season, when the exacerbation rate is higher [36]. Very few evidences support the effectiveness of montelukast combined to ICS as long-term asthma treatment or during an acute exacerbation [42,45].

### Montelukast and bronchiolitis

CysLTs are increased in respiratory secretions from infants with acute viral bronchiolitis and their levels remain significantly high at short-term follow-up, suggesting a possible role of CysLTs in the pathogenesis of the disease [46]. However, the results of the published RCTs are controversial. Amirav and coworkers demonstrated that montelukast given to infants with a first episode of bronchiolitis from the hospital admission until discharge did not reduce the length of stay, or the cytokine levels in nasal lavage fluid, nor improved the clinical severity scores [47]. Likewise, no significant benefit of montelukast administered both at 4 and 20 weeks after the onset of respiratory symptoms was shown in a large study of infants with post respiratory syncytial virus (RSV) bronchiolitis [48]. A small RCT confirmed this result, showing that montelukast given for three months after hospital admission for RSV bronchiolitis did not reduce the respiratory symptoms during both the treatment and the follow up periods [49]. All these findings appear conflicting with the conclusions of one of the first studies of RSV bronchiolitis that showed that montelukast results in significant improvement of symptoms score and in decrease of nighttime symptoms and daytime cough [50]. Finally, a recent RCT demonstrated that montelukast given for 3 months reduces eosinophilic degranulation (p < 0.01) and decreases the number of recurrent wheezing episodes (p = 0.039) in children 6 to 24 months-old with post-RSV bronchiolitis [51].

These conflicting results do not allow to reach definitive conclusions, and highlight the need for further studies on children with recurrent wheeze associated with RSV bronchiolitis.

### Acute asthma

The use of montelukast in acute asthma has received very poor attention. High concentrations of CysLTs have been found in urine patients with acute asthma exacerbations [52], and LTRAs administration could thus improve symptoms control during asthma attacks.

There are no studies on oral montelukast use in children under 1 year of life with acute wheezing. A RCT of 51 children aged 2 to 5 years with mild to moderate asthma evaluated the use of montelukast combined with albuterol at the first symptoms of an acute exacerbation [52]. When compared to patients treated with albuterol alone, subjects who received montelukast showed lower severity of symptoms 90 minutes after the onset of the exacerbation. This difference lasted up to four hours after the first symptoms, and a lower need for oral steroids was also reported. Treatment with montelukast initiated in children with intermittent asthma at the onset of upper respiratory infections (URI) or acute asthma, reduced symptoms (p = 0.049) and days off from school (p < 0.0001), but did not affect the use of asthma medications (p = 0.25) [33]. An additional RCT demonstrated that in moderate-to-severe intermittent wheezing a 7-days course of montelukast does not decrease oral corticosteroid use over a 12-month period, when compared with placebo or budesonide inhalation in addition to albuterol (p = 0.15) [40]. Despite the association between URI and onset of asthma has been repeatedly documented [1,33,36,37], a recent RCT of 300 preschool children with URI showed that a 12-week treatment with montelukast did not reduce the incidence of URI [53].

Given the controversial data available until now, we believe that further studies are urgently needed to support the treatment with LTRAs of preschool children with acute wheezing, especially in infants younger than one year.

Main results from all the studies conducted in children with preschool wheezing disorders treated with montelukast, alone or combined with ICS, are summarized in Table 1 and Table 2.

**Table 1:** Studies of preschool wheezing children treated with montelukast alone or with inhaled corticosteroids (ICS)

	**Author**	**Pros**	**Cons**
	**Bisgaard**[30]	Protection against cold air-induced reactivity	
	**Hakim**[22]	Reduced methacoline-induced reactivity	
**Wheezing (short-term**	**Straub**[31]	Reduced airway resistance and exhaled nitric oxide	
**montelukast monotherapy)**	**Straub**[32]	Improved lung function and symptom score	
	**Robertson**[33]	Reduced healthcare resource use, symptoms, time-off school/parent work	No effect on hospitalization rate, symptoms duration, β_2_ or steroids use
	**Szefler**[38]	No difference v*ersus* budesonide for	Higher rates of exacerbations
		- time to 1st additional asthma drug at 12 weeks	
		- time to 1st attack requiring oral steroid	
**Wheezing (long-term**	**Kooi**[39]	Montelukast *versus* fluticasone or placebo	
**montelukast**		In all groups	In all groups: no differences
**monotherapy**)		- Improved symptoms score	in lung function
		- Reduced blood eosinophils	
	**Allen-Ramey**[41]	Emergency visits fewer *versus* fluticasone	No differences in hospitalizations or rescue drugs
	**Davies**[35]	Similar rates of healthcare resource of cromolyn or ICS	
	**Bisgaard**[36]	Lower	
		- rate of asthma exacerbations	
		- median time to first exacerbation	
		- rate of ICS courses	
	**Bacharier**[40]		No difference in symptom free-days, oral steroid, healthcare resource use
**Wheezing**	**Johnston**	Reduced risk of :	
**(montelukast + ICS)**	[44]	- worsened asthma symptoms	
		- unscheduled physician visits	

References are in parenthesis.

**Table 2:** Studies of children with bronchiolitis and post-bronchiolitis, and acute asthma treated with montelukast

	**Author**	**Pros**	**Cons**
			No difference:
			- in length of stay
	**Amirav**[47]		- in clinical severity score
			- in cytokine levels in nasal lavage fluid
	**Bisgaard**[48]		No differences in percentage symptom-free days
			No differences:
**Bronchiolitis and post bronchiolitis**	**Proesmans**[49]		- of symptoms and disease-free days and nights
			- of n° of exacerbations
			- of n° of unscheduled visits and need of inhaled steroids
		Higher percentage of symptom-free days and nights	
	**Bisgaard**[50]	Reduced daytime cough	
		Decreased exacerbations *versus* placebo	
	**Kim**[51]	Reduced serum eosinophil-derived neurotoxin levels compared with initial levels	
		Decreased cumulative recurrent wheezing episodes at 12 months *versus* placebo	
	**Harmanci**[52]	Reduced oral steroids need	
**Acute asthma**		Decreased lung index scores and respiratory rate *versus* placebo	Hospitalization rates not significantly different
	**Robertson**[33]	Reduced healthcare resource use, symptoms, time-off school/parent work	No effect on hospitalization rate, symptoms duration, β_2_ or steroids use
	**Bacharier**[40]		No difference in symptom free-days, oral steroid, healthcare resource use

References are in parenthesis.

### Adverse events

LTRAs are generally well tolerated. Most of the adverse events described are mild (headache, gastrointestinal disorders, pharyngitis, cutaneous rash and reversible alterations in levels of serum transaminase) and do not significantly differ from those described in subjects receiving placebo [54].

Among respiratory medications used in the Italian pediatric population, montelukast is seldom prescribed as off-label drug, and its use is overall increasing [55]. This trend reflects recommendations from asthma guidelines, that support beneficial effects of LTRAs also in children aged less than 6 years [29]. A recent review on safety and tolerability of montelukast in children with both episodic (viral) and persistent multi-trigger wheeze concluded that its safety profile is comparable to that of placebo [54]. Adverse events most frequently reported include upper respiratory tract infections, worsening of wheeze, fever and self-limited cough, particularly in preschool children.

Risk for hepatic toxicity appears lower for montelukast than other molecules of the same group, and no increased risk of hypertransaminasemia during the treatment has been reported [45]. A large study on 1.948.297 children aged less than 5 years reported 3698 cases of montelukast overdosing, and authors concluded that no risk of relevant adverse events is associated to ingestion of more than 536 mg or 34 mg/kg [56]. No toxic effects have been described in two asthmatic children aged 2 and 5 years who were managed at home and in an emergency department, respectively, after unintentional poisoning [57]. This report concludes that montelukast does not entail toxic effects for doses below 4.5 mg/kg.

Churg-Strauss syndrome (CSS) is a rare but life-threatening granulomatous and eosinophilic vasculitis that occurs preferentially in school-age, adolescents and adults with pre-existing asthma [58]. Since the introduction of LTRAs, several studies reported the development of this condition after the onset of the treatment, particularly with zafirlukast [59], but CSS has never been described in preschool children.

The neuropsychiatric manifestations reported in old children during montelukast treatment have never been observed in preschool age. Nevertheless, even in adults and adolescents, the results of the clinical trials do not provide substantial evidence to support a causal association between montelukast therapy and such disorders [60].

All the above reported data support a good safety profile of montelukast and encourage its use in the clinical practice. Future long-term studies in preschool children will provide further information regarding potential adverse events of LTRAs use in this age range.

### From asthma guidelines to recommendations

The main guidelines on asthma management (Global Initiative for Asthma, GINA; British Thoracic Society, BTS; National Institute of Health, NIH) have confirmed the clinical utility of LTRAs in children older than five years [15-17]. In school age children and adolescents, LTRAs are recommended in mild persistent asthma as second-line therapy in alternative to ICS, or in moderate persistent asthma as add-on to ICS, in alternative to ICS alone [17] with beneficial effects on asthma control and on the number of exacerbations [16,61]. Moreover, regular treatment with LTRAs effectively protects against exercise-induced bronchoconstriction, with no tolerance to the bronchoprotective effect [23].

In children younger than 5 years of age, only very few RCTs have been conducted, and therefore the recommendations made by official guidelines on LTRAs treatment mainly derive from studies conducted in older children. In the GINA guideline LTRAs are suggested especially because of their efficacy in reducing virus-induced exacerbations, which are extremely frequent at preschool age [15]. According to the BTS document, LTRAs may be administered in alternative to ICS, or as an add-on therapy in children who do not fully respond to ICS [16]. Finally, the NIH guideline recommends LTRAs to young children to overcome the difficulties due to the use of inhalator devices, or when compliance is poor [17].

In 2008 two relevant documents have provided indications for asthma management in preschool age [13,62]. First, the PRACTALL (“PRACTicing ALLergology”) consensus report concluded that LTRAs are effective as short- or long-term therapy for controlling virus-induced asthma exacerbations in children under 2 years of age, and may be used alone in alternative to ICS in children 3 to 5 years old with intermittent or mild persistent asthma, [13]. Second, a Task Force committed by the European Respiratory Society (ERS) identified two different wheezing phenotypes: a) episodic (viral) wheezing, characterized by intermittent wheeze and no symptoms between episodes; and b) multiple-trigger wheeze, induced by several stimuli including exercise and allergens, in children who wheeze both during and outside discrete episodes [62]. Indeed, this classification has raised several concerns. The identification of the wheezing phenotype is generally based on symptoms reported by parents, and a wide overlapping of clinical symptoms and signs between the two categories may occur [63]. Moreover, preschool wheezing phenotypes can change over time, entailing relevant modifications in previously established treatment decisions [64]. As far as LTRAs, the ERS document concluded that a trial with montelukast may be considered in both multi-trigger and viral wheeze, but that further studies are needed to strengthen these recommendations [62].

Important keypoints about montelukast in preschool wheezing are summarized in Table 3. They include both evidences definitively supporting its use in the clinical practice, and uncertainties that still limit its prescription.

**Table 3:** Keypoints in the use of montelukast in preschool children with wheezing disorders

**Evidences**	**Controversies**
Effectiveness in episodic (viral) wheeze	Effectiveness of intermittent use in severe recurrent wheeze
Effectiveness in multi-trigger wheeze	Effectiveness of combined therapy with inhaled steroids in wheezing children
Reduction of airway inflammation	Effectiveness in severe post-RSV bronchiolitis wheeze
Excellent safety profile	Effectiveness in acute exacerbation
Good compliance due to oral single administration	Selection criteria of subjects with wheezing to treat with monotherapy or combined therapyv with inhaled steroids

## Conclusions

The optimal therapeutic strategy for wheezing disorders in early life remains elusive. The pathophysiology is still poorly understood, and likely includes factors other than airway inflammation [65]. Future research in this area should hopefully incorporate consideration in particular of the heterogeneous nature of asthma in preschool children.

Since current recommendations on the use of montelukast, both alone or combined with ICS, derive from evidences in older children, further research is urgently needed to confirm the effectiveness of LTRAs in young wheezing children. Based on the current body of evidence, there is rationale for further investigation of these management strategies, including direct comparisons between ICS and LTRAs, as well as the role of long-acting beta-agonists, potentially targeting the subpopulations of early wheezers who are at highest risk for the persistence of asthma symptoms.

An additional important keypoint is represented by the identification of subjects who wheeze and may benefit from montelukast alone, or from combined therapy. In particular, combined therapy with ICS *plus* LTRAs might result effective in achieving symptom control and in decreasing health care resource utilization [66]. The intermittent use of montelukast for treating in particular preschool children with severe recurrent wheeze should be more investigated. Finally, further studies should be promoted to confirm that montelukast is effective in preschool acute asthma [53] and to clarify the controversial data published on LTRAs effects in post-RSV bronchiolitis [37,48,49].

Montelukast is a safe drug, with a prominent anti-inflammatory activity to the airways [31], and also a strong bronchoprotective effect [30]. Montelukast administered as inhaled drug has been recently reported to provide a significant bronchodilation compared to placebo, in adolescents and adults with chronic asthma [67], but no studies are available in school-age or preschool children.

The excellent safety profile of montelukast, and the possibility of oral administration, which entails better compliance from young children, represent the main strengths of its use in preschool children. Therefore, montelukast represents a valid alternative to ICS in poorly compliant children, or in subjects who show adverse effects related to long-term steroid therapy.

## Abbreviations

ICS: Inhaled corticosteroids; LTRAs: LTs, Leukotrienes; LTRAs: Leukotriene receptor antagonists; CysLTs: Cysteinyl leukotrienes; GINA: Global Initiative for Asthma; BTS: British Thoracic Society; NIH: National Institute of Health; RCT: Randomized controlled clinical trial; ERS: European Respiratory Society; RSV: Respiratory syncytial virus.

## Competing interests

All authors declare that they have no significant competing financial, professional or personal interests that might have influenced the performance or presentation of the work described in this manuscript.

## Authors’ contributions

SM has been involved in drafting the manuscript and revising it critically for important intellectual content; MM has been involved in drafting the manuscript; SDS has been involved in drafting the manuscript; AM has made substantial contributions to acquisition, analysis and interpretation of data; ADG has made substantial contributions to acquisition, analysis and interpretation of data; FS has been involved in drafting the manuscript, revising it critically for important intellectual content and has given final approval of the version to be published. All authors read and approved the final manuscript.
